# Growth inhibitory effect of selected medicinal plants from Southern Ethiopia on the mycelial phase of *Histoplasma capsulatum* var. *farciminosum*

**DOI:** 10.1186/s12917-023-03873-0

**Published:** 2024-01-19

**Authors:** Tagash Girma, Gemechu Chala, Berhanu Mekibib

**Affiliations:** Faculty of Veterinary Medicine, Hawassa University, Hawassa, Ethiopia

**Keywords:** Epizootic lymphangitis, Hawassa, Histoplasma, In vitro, Medicinal Plants, Mycelia

## Abstract

**Background:**

Epizootic lymphangitis is an infectious and chronically debilitating disease of the equines. *Histoplasma capsulatum* var. *farciminosum,* a thermally dimorphic fungi, is the causative agent for the disease. In Ethiopia, the disease significantly affects carthorses, posing threats to animal welfare, and resulting in substantial economic losses. Limited availability of widely accessible antifungals in addition to the chronic nature of the disease is the major challenge against management of epizootic lymphangitis. This study aimed to assess the in vitro efficacy of specific local medicinal plant extracts against the mycelial phase development of *H. capsulatum var. farciminosum* in southern Ethiopia. The leaves of *Xanthium strumarium*, *Kanda* (Family *Rubiaceae*), *Croton macrostachyus* (*Bisana* in Amharic), and *Centella Asiatica* (*Echere waye* as a local name in Zeyissegna) that are traditionally used for the treatment of different skin ailments were collected and extracted for the in vitro trial.

**Results:**

The study revealed that methanol extracts of *Xanthium strumarium*, *Kanda*, *Croton macrostachyus*, and *Centella Asiatica*, at minimum inhibitory concentrations of 1.25 mg/ml, 2.5 mg/ml, 2.5 mg/ml, and 5 mg/ml, respectively, inhibited the growth of *H. capsulatum var. farciminosum*.

**Conclusion:**

This in vitro finding could serve as significant preliminary data in the exploration of effective alternative treatment options for epizootic lymphangitis. This study provides a crucial foundation for further research aimed at determining the chemical components and in vivo effectiveness of these plant extracts against both the mycelial and yeast forms of *Histoplasma capsulatum* var. *farciminosum*.

## Background

In Ethiopia, equines play a crucial role in transportation services, particularly in rural areas with limited access to motorized transportation [1]. Unfortunately, the welfare of these animals is severely compromised due to poor management and diseases such as ulcerative lymphangitis and epizootic lymphangitis [1–3]. Epizootic lymphangitis, a contagious and chronically debilitating disease, is caused by *H. capsulatum var. farciminosum*, a thermally dimorphic fungus existing as a yeast form in animal tissue and a saprophytic or mycelial form in the environment [4–6].

The incidence of epizootic lymphangitis is most prevalent in tropical and subtropical regions, thriving in sub-Saharan Africa, notably in Ethiopia, and documented in northern and northeastern Africa, parts of Asia, Japan, India, Pakistan, and countries bordering the Mediterranean Sea [4, 7, 8]. The clinical symptoms include ulcerating and spreading pyogranulomatous, suppurative, multifocal dermatitis, and lymphangitis [4, 9]. Unfortunately, the disease is poorly responsive to available drug therapies, demanding prolonged treatment, often unaffordable for many animal owners [3, 4, 10–12], leading to uncontrollable spread, significant economic loss, and adverse impacts on livelihoods in endemic areas like Ethiopia [10, 13–15].

Epizootic lymphangitis is widespread in Ethiopia with a prevalence ranging from 13.33–39.1% [1, 8, 16–18] and affecting equines used for cart pulling, posing a significant threat to households relying on this business [3, 10, 16, 19–21]. Cart-pulling equids are particularly vulnerable due to untreated harness-related skin damage, predisposing them to infection. Owners, faced with the unavailability or unaffordability of effective treatments, often continue using their animals until they become severely affected and unable to work, leading to abandonment [22].

Globally, there is growing interest in exploring alternative approaches to treating various ailments, with a focus on medicinal plants [23]. In Ethiopia, several medicinal plants, including *Aloe* species, *Eucalyptus globulus*, *Hagenia abyssinica*, *Cupressus macrocarpa*, *Acmella caulirhiza*, *Buddleja polystachya*, *Acacia* species, *Clematis species*, *Croton macrostachyus*, and *Moringa stenopetala*, have been studied with promising results regarding their inhibitory effects on *H. capsulatum var. farciminosum* growth [20, 24, 25]. Despite traditional use, more needs to be learned, making in vitro evaluations crucial to understanding the pharmacodynamic activities and disease management potential of these plants [23, 26, 27].

In southern Ethiopia, leaves of plants like *Xanthium strumarium*, *Croton macrostachyus* (*Bisana*), *Centella Asiatica*, and *Kanda* (from the *Rubiaceae* family) have been traditionally used for treating various animal ailments, including wounds, bacterial, and fungal diseases like epizootic lymphangitis. While this traditional knowledge is valuable, scientific validation is lacking. Despite their wider use among the local households, however limited or no studies have explored the antimicrobial properties, active ingredients, and minimum inhibitory concentration values of these plants in the country. This study aims to demonstrate the growth inhibition effect of the leaves of *Xanthium strumarium*, *Croton macrostachyus* (*Bisana*), *Centella Asiatica*, and *Kanda* (from the *Rubiaceae* family) on the mycelial form of *H. capsulatum var. farciminosum* isolates found in active cases of epizootic lymphangitis in southern Ethiopia.

## Results

### Occurrence of epizootic lymphangitis

A clinical examination was conducted on 581 equines, comprising 191 horses, 127 mules, and 263 donkeys, to identify the presence of epizootic lymphangitis lesions. Both clinical and mycological assessments were performed, revealing that 7.1% (41/581) of the animals were affected by the disease. Among the positive cases, 27 (14.1%; confidence interval [CI]: 2.85%—17.45%) were horses, 8 (6.3%; CI: 0.98%—8.48%) were mules, and 6 (2.3%) were donkeys.

**Fig. 1:**
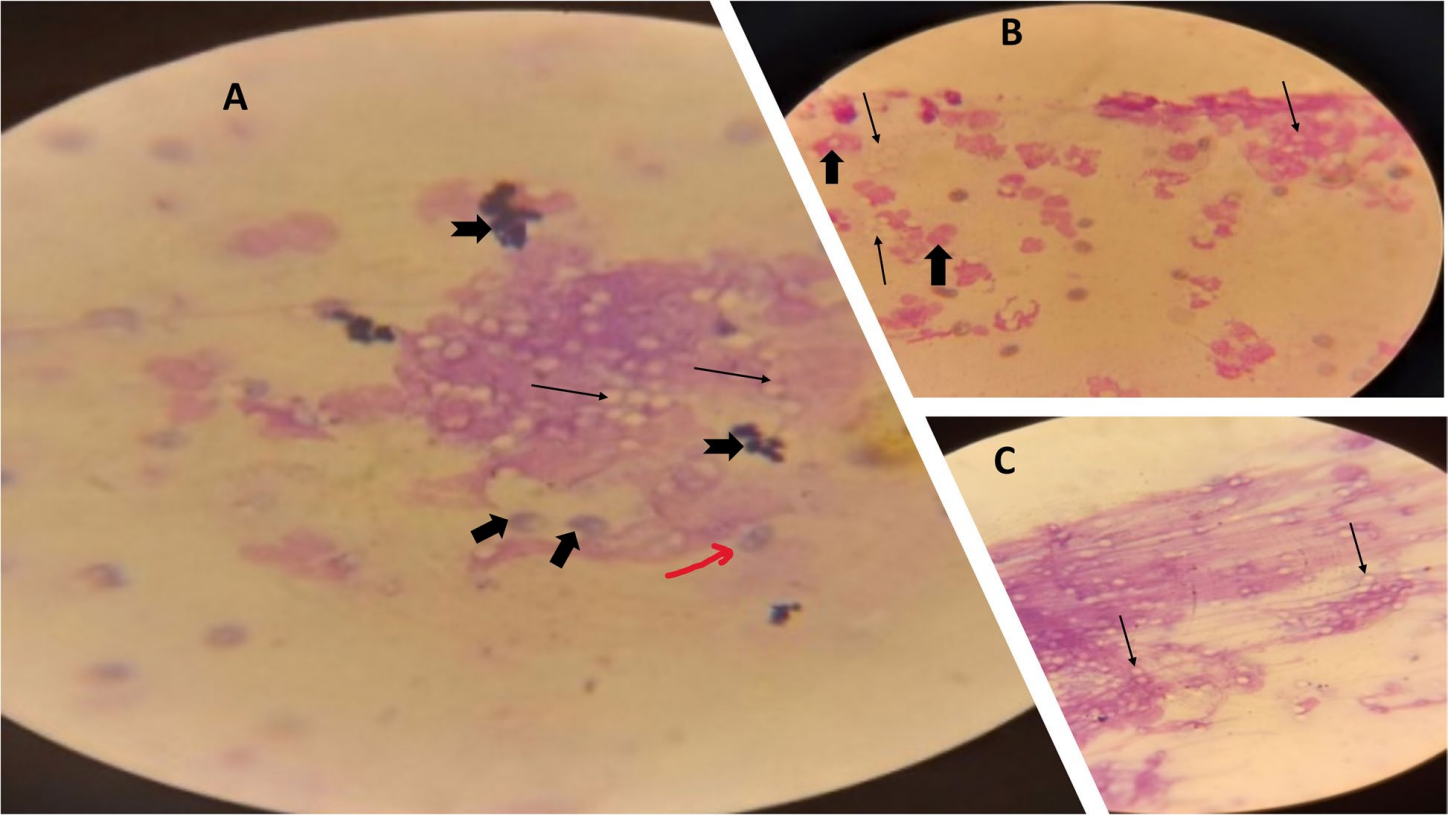
Characteristic *Histoplsma capsulatum* variety *farciminosum* yeast cells from clinical cases stained with Wright stain: (**A**) from Mid (black thin arrows shows yeast cells; flat arrows indicate macrophage engulfed yeast cells; flat and indented arrows shows neutrophils whereas red arrow indicates budding yeast cell); (**B**) Early (flat arrows indicate macrophage engulfed yeast cells whereas thin arrows indicate yeast cells and (**C**) advanced stages of the disease (arrows indicating yeast cells)

**Fig. 2:**
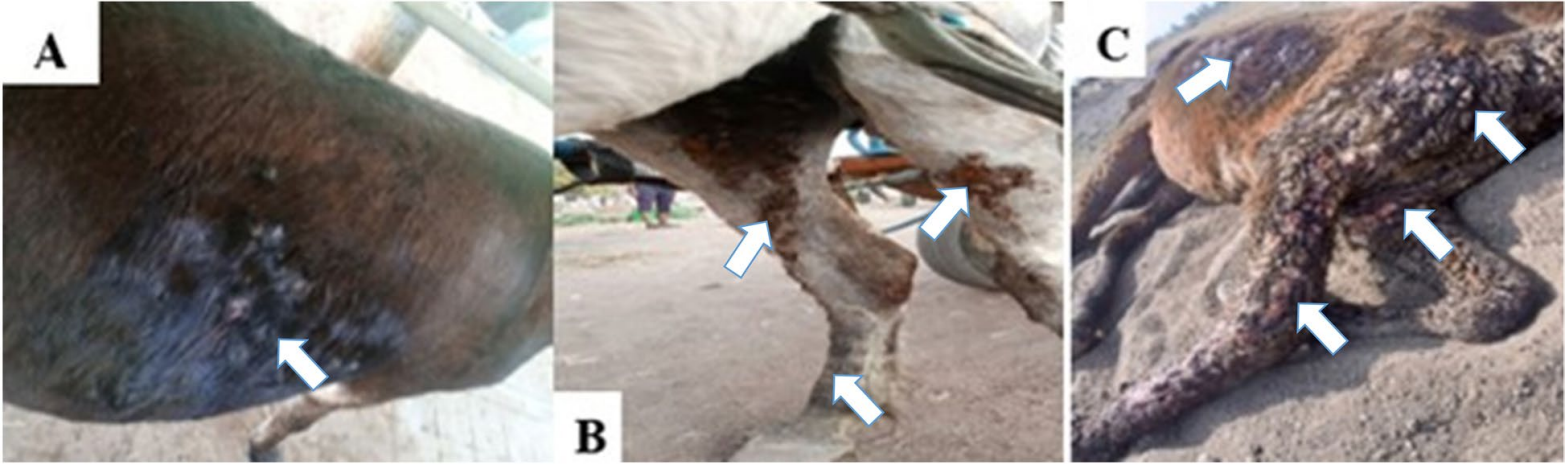
Characteristic cutaneous epizootic lymphangitis lesion seen during the study: (**A**) donkey; (**B**) horse and (**C**) mule. Arrows showing ulceration, cording of the lymphatics and the skin with fistulation (in **A**) and ulcerating nodules as in **B** and **C** where most of the nodules coalesced

Microscopic examination of Wright/Giemsa-stained smears obtained from clinical epizootic lymphangitis samples revealed the typical yeast form of *H. capsulatum var. farciminosum*, characterized by ovoid aggregates surrounded by a halo (Fig. 1). Additionally, all observed clinical cases of epizootic lymphangitis were exclusively limited to the cutaneous form of the disease (Fig. 2).

### Isolation and identification of *H. capsulatum var. farciminosum*

To confirm the presence of *H. capsulatum var. farciminosum* in cases of epizootic lymphangitis, samples were collected and examined using Wright/Giemsa-stained smears. Positive samples were then cultured on Sabouraud's dextrose agar (SDA) and incubated at 26.8 °C for 12 weeks. The SDA was inspected every two weeks to monitor any growth of *H. capsulatum var. farciminosum*.

**Fig. 3:**
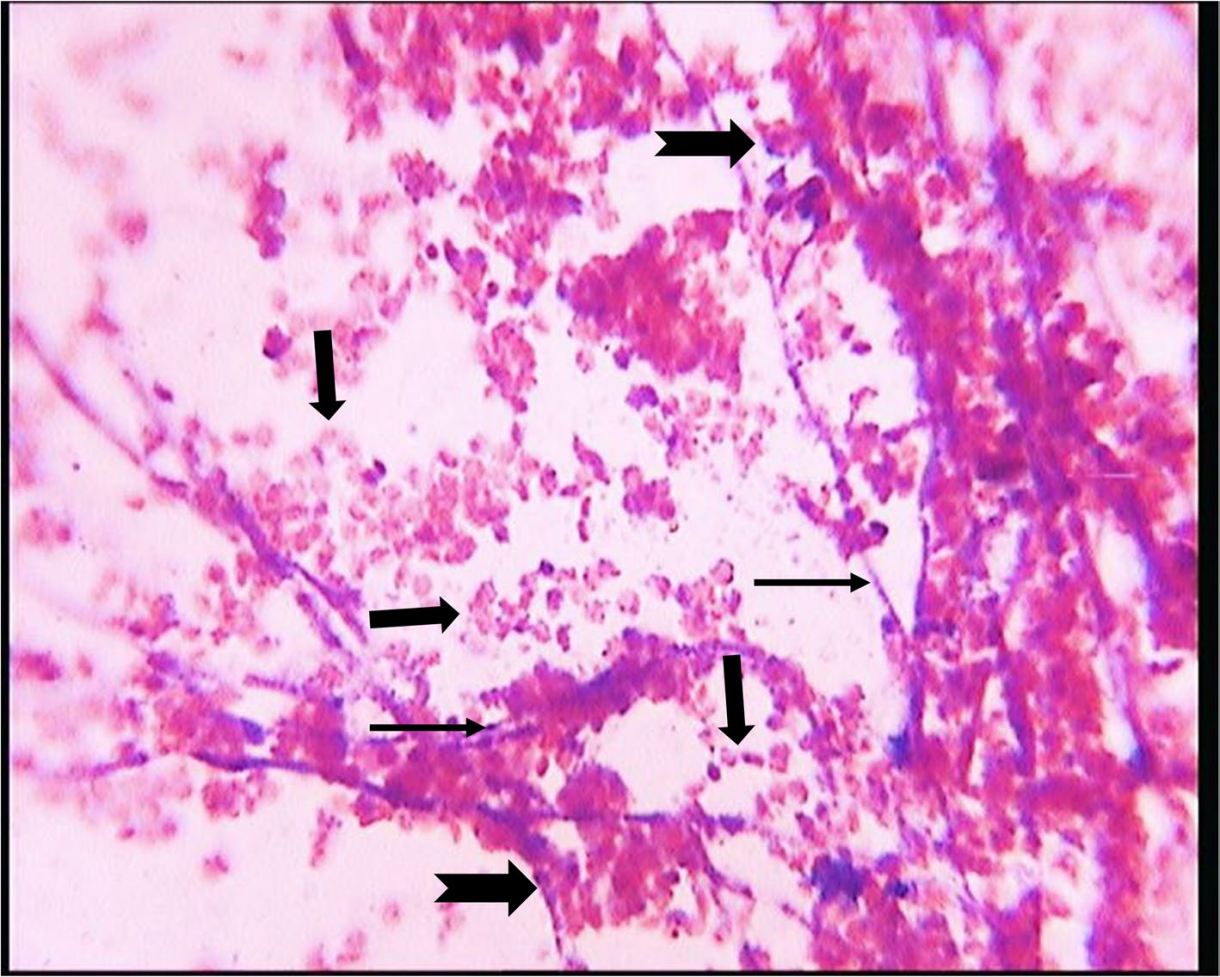
Microscopy of Gram-stained smears of *Histoplsma capsulatum* variety *farciminosum* from 12th weeks of incubation (multiple small arrows indicating a septate hyphae bearing rounded (sometimes speculated) macroconidia whereas indented flat arrows show microconidia

During the initial 1–5 weeks of incubation, whitish, foamy, cotton-like colonies of H. capsulatum var. farciminosum were observed. Subsequently, between 6–8 weeks, these colonies underwent a transformation in appearance, becoming folded, waxy, and ranging in color from gray to yellowish-brown. Presumptive colonies after 12 weeks of incubation were identified through Gram-stained culture smears, revealing a septate hyphae bearing rounded (sometimes speculated) macroconidia and microconidia (as illustrated in Fig. 3).

### Results of in vitro* H. capsulatum var. farciminosum* growth inhibition effects of the plants

**Fig. 4:**
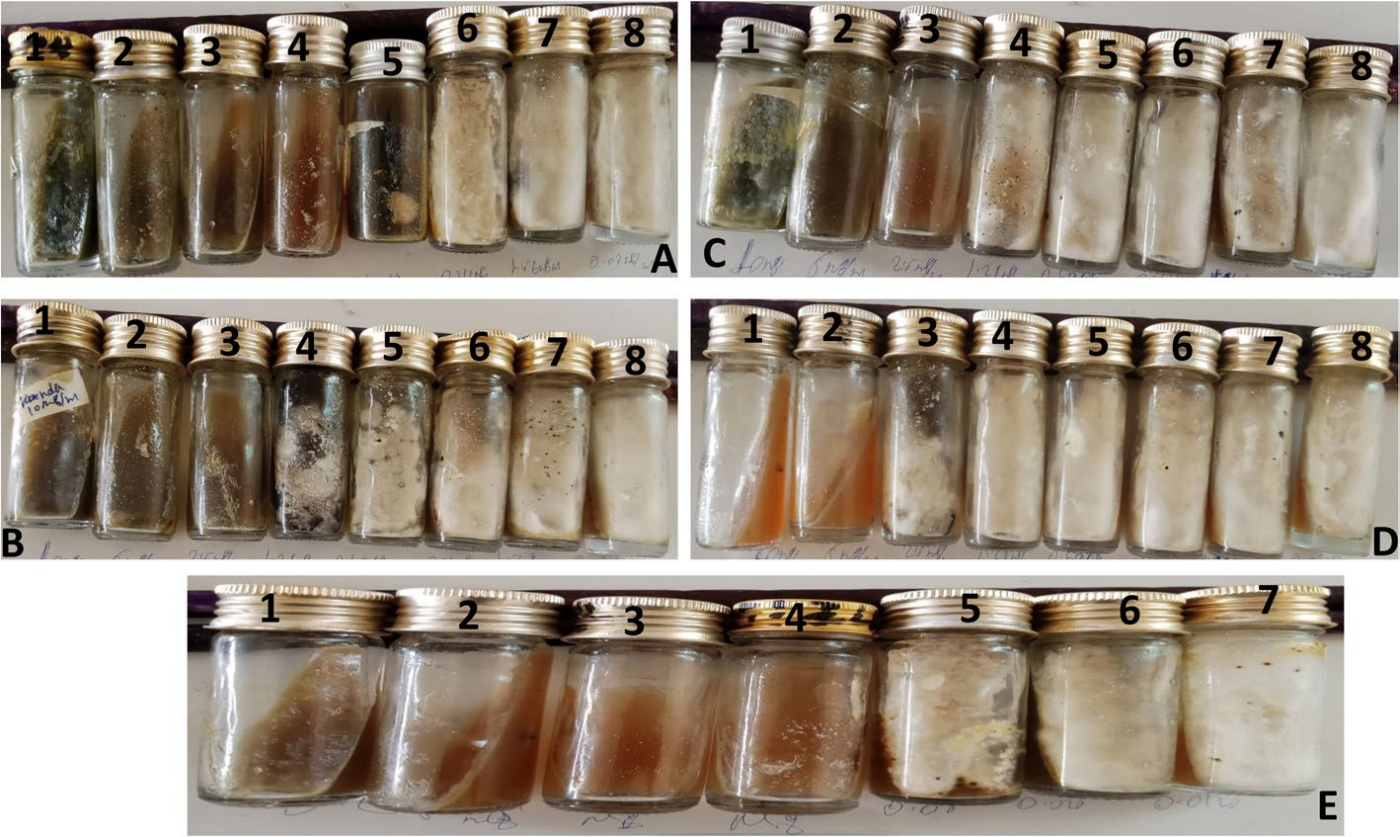
Pictorial representation of growth inhibitory effects of *Xanthium strumarium* (**A**), *Kanda* (**B**), *Croton macrostachyus* (**C**), *Centella Asiatica* (**D**), and positive control (ketoconazole) (**E**) at different concentrations. The 1–8 numbers (from **A** to **D**) indicates plant extract concentrations ranging from 10 mg/ml, 5 mg/ml, 2.5 mg/ml, 1.25 mg/ml, 0.625 mg/ml, 0.3125 mg/ml, 0.156 mg/ml, and 0.78 mg/ml, respectively whereas the 1–7 numbers (**E**) indicate the Ketoconazole concentration ranging from 0.8 mg/ml, 0.4 mg/ml, 0.2 mg/ml, 0.1 mg/ml, 0.05 mg/ml, 0.025 mg/ml, and 0.0125, respectively

Table 1 presents the qualitative evaluation results of the growth inhibition effect of *H. capsulatum var. farciminosum* Different leaf extracts from various plants were tested against selected representatives of *H. capsulatum var. farciminosum* isolates obtained from culture-positive clinical samples. The results highlighted that the methanol extract from *Xanthium strumarium* demonstrated a notable anti-*H. capsulatum var. farciminosum* effect. This extract exhibited a minimum inhibitory concentration of 1.25 mg/ml, inhibiting *H. capsulatum var. farciminosum* growth at concentrations ranging from 1.25 mg/ml to 10 mg/ml (Fig. 4A).

**Table 1:** In vitro evaluation of growth inhibitory effects of methanol extracts of selected medicinal plants on *Histoplasma capsulatum* var. *farciminosum*

*X. strumarium*		*C. macrostachyus*		*Kanda*		*C. asiatica*		*Ketoconazole*	
Conc. (mg/ml)	Growth	Conc. (mg/ml)	Growth	Conc. (mg/ml)	Growth	Conc. (mg/ml)	Growth	Conc. (µg/ml)	Growth
10	X	10	X	10	X	10	X	0.8	X
5	X	5	X	5	X	5	X	0.4	X
2.5	X	2.5	X	2.5	X	2.5	-	0.2	X
1.25	X	1.25	-	1.25	-	1.25	-	0.1	X
0.625	-	0.625	-	0.625	-	0.625	-	0.05	-
0.3125	-	0.3125	-	0.3125	-	0.3125	-	0.025	-
0.156	-	0.156	-	0.156	-	0.156	-	0.0125	-
0.078	-	0.078	-	0.078	-	0.078	-	0.00625	-

Keys: (X) = Growth inhibited; (-) Growth not inhibited

Similarly, methanol extracts from *Kanda* and *Croton macrostachyus* also displayed promising anti-*H. capsulatum var. farciminosum* effects, inhibiting growth at concentrations ranging from 2.5 mg/ml to 10 mg/ml (Fig. 4B and C). Additionally, *Centella Asiatica* methanol extract showed a minimum inhibitory concentration of 5 mg/ml, and its anti-*H. capsulatum var. farciminosum* effect was observed at concentrations ranging from 5 mg/ml to 10 mg/ml (Fig. 4D). It's noteworthy that positive (drug) control, Ketoconazole demonstrated a minimum inhibitory concentration of 0.1 µg/ml. On the contrary, negative controls revealed that *H. capsulatum* var. *farciminosum*, when cultured on antifungal-free SDA, exhibited sustained growth (growth control), while no fungal growth was evident on non-inoculated antifungal-free SDA (no-growth control) (Fig. 4E).

## Discussion

Horses play a vital role in rural transportation, yet they often suffer from diseases such as epizootic lymphangitis, impacting their well-being and productivity with subsequent economic implications [16]. Treatment options for this disease are limited, especially in resource-limited countries like Ethiopia. Researchers are actively seeking effective treatment and management strategies. This study investigated the growth-inhibiting properties of certain plant extracts, including *X. strumarium*, *Kanda*, *C. macrostachyus*, and *C. Asiatica*, on the mycelia of *H. capsulatum var. farciminosum* using qualitative methods.

The study found that 7.1% of horses used for cart-pulling were diagnosed with epizootic lymphangitis, with a prevalence lower than previous studies in the same area [1] but higher than others [16]. Variations could be attributed to differences in sample size, study method, agroecology, and study period. The larger sample size in this study, including resistant species like mules and donkeys, may have contributed to the lower prevalence observed.

The investigation into the inhibitory effects of certain medicinal plants on the growth of *H. capsulatum var. farciminosum* yielded optimistic results. Extracts from *X. strumarium*, *Kanda*, *C. macrostachyus*, and *C. Asiatica* leaves demonstrated promising inhibitory effects on the mycelial growth of *H. capsulatum var. farciminosum*, with concentrations ranging from 1.25 mg/ml to 10 mg/ml. While Ketoconazole proved more effective, the plant extracts, especially *X. strumarium*, exhibited comparable inhibitory effects on *H. capsulatum var. farciminosum* mycelial growth. *X. strumarium*, with a minimum inhibitory concentration (MIC) of 1.25 mg/ml, demonstrated potent anti-*H. capsulatum var. farciminosum* effects, aligning with prior research [5]. Phytochemical analysis revealed antibacterial and antifungal substances in *X. strumarium*, supporting its traditional use for treating various conditions [5, 37].

*C. macrostachyus* showed noteworthy antifungal properties against *H. capsulatum var. farciminosum* mycelia. The plant has a rich history of medicinal use for various ailments, supported by its antibacterial, antifungal, anthelmintic, anti-inflammatory, antidiarrheal, and antioxidant properties [34–36]. The study suggests that this result could be promising data for the exploration of effective treatment options against *H. capsulatum var. farciminosum* infection.

*C. Asiatica* and *Kanda* methanol extracts showed significant anti-*H. capsulatum var. farciminosum* effects, with MIC values of 5 mg/ml and 2.5 mg/ml, respectively. While not previously reported for *H. capsulatum var. farciminosum*, both plants are known for their neuro-protective, antifungal, antibacterial, anti-diabetic, and antioxidant properties [38, 39]. Phytochemical composition, including saponins, glycosides, tannins, essential acids, phytosterols, mucilage, resins, free amino acids, flavonoids, a bitter component (valerene), and fatty acids in *C. Asiatica*, may contribute to its effectiveness against *H. capsulatum var. farciminosum* [40, 41].

Although *Kanda* lacks written documentation for its effects against *H. capsulatum var. farciminosum*, traditional use in the Zeyisse community in Ethiopia indicates its efficacy against ringworm and parasitic infections in both humans and animals.

Further research is needed to validate these findings, yet the study provides promising evidence of targeting these plants as potential candidates in future research on exploration of effective treatment options for epizootic lymphangitis, addressing the current lack of effective, affordable, and universal treatments.

The current study exclusively examined the impact of methanol extracts from the leaves of *X. strumarium*, *Kanda*, *C. macrostachyus*, and *C. Asiatica* plants on the in vitro growth of the mycelial form of *H. capsulatum var. farciminosum* It is crucial to acknowledge that this form possesses different growth characteristics and susceptibility profiles than its yeast form [27, 42]. Therefore, further research is essential to thoroughly investigate the inhibitory effects of these plants on the growth of the yeast form of *H. capsulatum var. farciminosum* Additionally, exploring the in vivo effects on both the mycelial and yeast forms of the fungi will provide a more comprehensive understanding of the therapeutic potential of these plant extracts.

## Conclusion

Our in vitro assessment clearly indicates that the methanol extracts of *X. strumarium*, *Kanda*, *C. macrostachyus*, and *C. Asiatica* leaves exhibit a substantial inhibition of the growth of mycelial forms of *H. capsulatum var. farciminosum*. This suggests that these plants may represent a valuable resource in the quest for new treatment options for epizootic lymphangitis—a chronic disease with significant economic implications, especially in countries like Ethiopia. Our future research endeavors will focus on determining the growth inhibitory effect of these plants against both the yeast and mycelial forms of *H. capsulatum var. farciminosum* through in vitro and in vivo studies. Additionally, it is crucial to identify and purify the active ingredients present in these plant extracts. Despite its limitations, this study could mark a significant milestone in the treatment and control of epizootic lymphangitis.

## Methods

### Study design, study population, and sample size determination

A cross-sectional study was conducted from February 2022 to April 2023 to collect and analyze samples from cart-pulling equines found in Hawassa City, southern Ethiopia, and in vitro trial was conducted at Hawassa University, Veterinary Microbiology laboratory to evaluate the growth inhibitory effect of selected medicinal plants on HCF. Hawassa city, which is the capital for both Sidama and Southern nations, nationalities, and peoples of Ethiopia is in the major Rift Valley at about 275 km south of Addis Ababa [28]. This study considered all cart-pulling equine populations found in the city. A single population proportion formula [29] was used to compute the required sample size using a 95% confidence level, 5% desired absolute precision and 13.33% previous prevalence [1] as follows:

$$\mathrm n\:=\:\left(1.96\right)^2\;\left[\mathrm{Pexp}\left(1-\mathrm{Pexp})\right)\right]/\mathrm d^2$$

Where: *n* = required sample size,

$$\mathrm{Pexp}=\;\mathrm{expected}\;\mathrm{prevalence},\;$$

$$\mathrm d^2\;=\mathrm{desired}\;\mathrm{absolute}\;\mathrm{precision}.$$

Accordingly, the calculated sample size was 178. However, to increase the study precision, increase the probability of capturing clinical cases of the disease, and avoid unnecessary loss of study subjects, the final sample size was increased to 581. So, during the study period a total of 263, 191, and 127 donkeys, horses, and mules, respectively, were considered.

### Sampling method and sample collection

Carthorses and mules were conveniently sampled due to their limited numbers in the city, while cart donkeys were randomly sampled, given their larger population. Clinical samples (pus) were aspirated using a syringe and needle from apparently unruptured nodules into a sterile sampling bottle. Before sample collection, the area was cleaned with water and soap, shaved with a scalpel blade, and disinfected with 70% ethanol. The collected samples were properly labeled and transported in a cool box with an ice pack to the Veterinary Microbiology Laboratory at Hawassa University, where mycological examination was conducted.

### Study methodology

#### Clinical examination of the study animals

Prior to the collection of mycological samples, each selected animal underwent a comprehensive clinical examination to identify the presence of characteristic lesions associated with epizootic lymphangitis. Animals were clinically presumed to be positive for epizootic lymphangitis when characteristic lesions, including the presence of nodules and ulcers, were observed. Samples for mycological investigation were then collected from these clinically positive animals and shipped to the Hawassa University Veterinary Microbiology Laboratory.

### Isolation and Identification of Histoplasma capsulatum var. farciminosum

Isolation and identification of *H. capsulatum var. farciminosum* was done according to the procedures and protocols described by [4]. Prior to culture, *H. capsulatum var. farciminosum* was initially identified by examining its morphological features under an oil-immersion microscope at × 100 magnification, using Wright and/or Giemsa-stained dried aspirate smears [30]. All samples that tested positive in the microscopic examination were then inoculated in Sabouraud’s dextrose agar (SDA) enriched with 2.5% glycerol for the isolation of *H. capsulatum var. farciminosum*. To inhibit the growth of bacterial contaminants, a chloramphenicol supplement (0.005%) was added to the culture media.

The inoculated media were incubated at 26.8 °C for 2 to 8 weeks, with regular weekly checks for the growth of the mycelial form of *H. capsulatum var. farciminosum* (indicated by the presence of dry, grey-white, granular, wrinkled colonies) [4, 31]. Presumptive colonies were subjected to Gram staining for microscopical confirmation of the morphological features of *H. capsulatum var. farciminosum* [32]. Microscopically confirmed *H. capsulatum var. farciminosum* isolates were then utilized for in vitro analysis of selected medicinal plant extracts against the growth of the organism.

### Preparation of the plant extracts

**Fig. 5:**
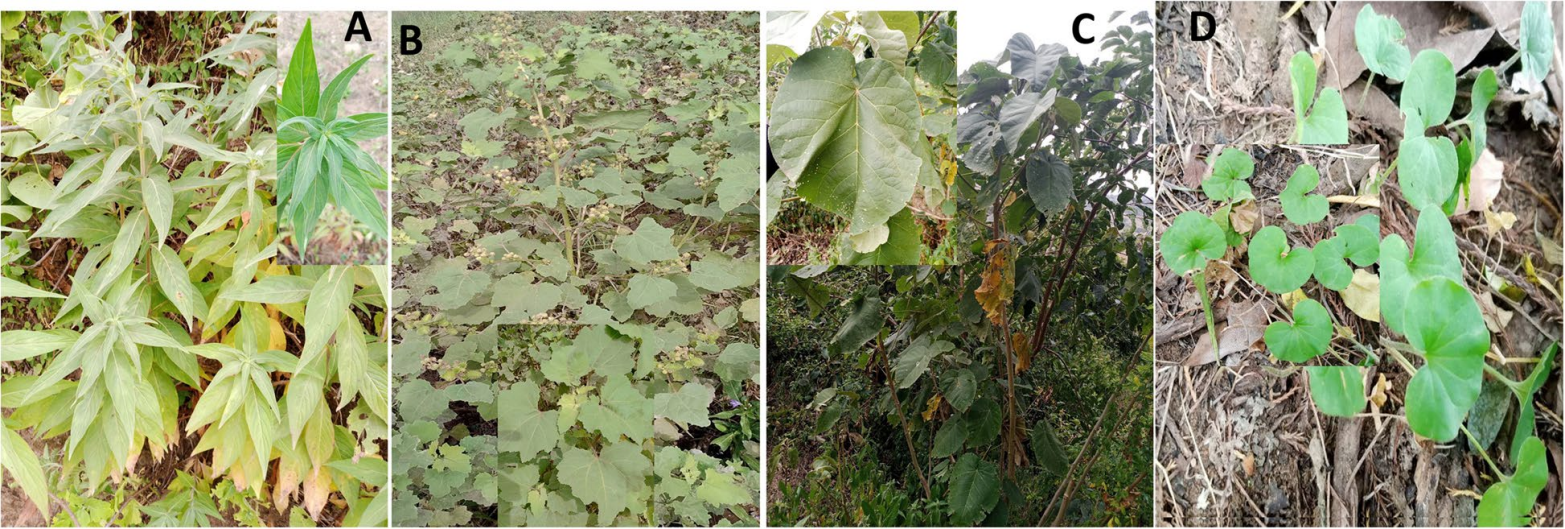
Portrayal of plants used in this study: (**A**) *Kanda* (family *Rubiaceae*); (**B**) *Xanthium strumarium*; (**C**) *Croton macrostachyus* (*Bisana)*; *and* (**D**) *Centella Asiatica (Echere waye)*

Four medicinal plants (Fig. 5), commonly employed in the local treatment of various fungal skin diseases such as dandruff, pityriasis versicolor, tinea corporis in humans, and ringworm in animals, were collected from diverse fields and gardens for screening their growth inhibition effects on *H. capsulatum var. farciminosum.* The plants were identified at Hawassa University by senior botanists. Subsequently, the leaves of *Xanthium strumarium* (Dhiha nikel in the local language), *Croton macrostachyus* (*Bisana* in the local language), *Centella Asiatica* (*Echere waye* in the local language), and *Rubiaceae* (*Kanda* in the local language) were air-dried under shade and manually ground into powder using a mortar and pestle.

The powdered leaves of *Xanthium strumarium*, *Rubiaceae*, *Croton macrostachyus*, and *Centella Asiatica* were then combined with 80% methanol at respective ratios of 1:5, 1:10, 1:8, and 1:5. The extraction process involved keeping the mixture on an orbital shaker for 48 h. The crude extract underwent filtration first with gauze, followed by Whatman filter paper (Number 1, diameter 6 mm; Whatman Ltd, England) into a sterile beaker. Methanol was subsequently evaporated from the filtrate using a vacuum rotary evaporator at 40 °C, and the extract was further dried in a microwave oven at 40 °C for one week to ensure complete drying. The resulting dried stock powder was stored at -4 °C until its use [12, 33].

### Preparation of testing solution

Serial dilution of the extracts began by dissolving 1000 mg of the plant extract in 10 ml of pure dimethyl sulfoxide (DMSO), followed by filtration with filter paper (Whatman No. 1). The filtrate was then re-diluted using sterile distilled water. Final concentrations of 10 mg/ml, 5 mg/ml, 2.5 mg/ml, 1.25 mg/ml, 0.25 mg/ml, 0.312 mg/ml, 0.156 mg/ml, and 0.07 mg/ml of the extracts were prepared for all the plants.

As a standard treatment (positive (drug) control), ketoconazole was diluted in distilled water at a recommended concentrations of 0.8 µg/ml, 0.4 µg/ml, 0.2 µg/ml, 0.1 µg/ml, 0.05 µg/ml, 0.025 µg/ml, and 0.0125 µg/ml. Negative controls included antifungal-free SDA inoculated with a fungal colony (growth control) and un-inoculated SDA (no-growth control), which were then incubated for further analysis [3, 26].

### Preparation of inoculums and inoculation of the media

Before inoculation, each concentration of the plant extracts was added to separate sterile bottles. An equal amount (9 ml) of already prepared Sabouraud’s dextrose agar (SDA) was added to each of the bottles containing plant extracts and allowed to solidify in a slant position. Previously identified mycelial colonies of *H. capsulatum var. farciminosum* were scraped from the SDA using a sterile inoculating loop and transferred to a sterile saline (0.85%) solution. We used five slants of Sabouraud’s dextrose agar for each concentration of the plant’s extracts. The mycelia were mixed by vortex until their turbidity matched the 0.5 McFarland standard, after which they were uniformly streaked onto each of the slants with the help of a sterile inoculating swab.

Finally, the lids of the inoculated agar plates were closed, and the plates were incubated at 26.8 °C for 4 weeks with weekly checkups for growth. At the end of the 4-week incubation period, the tubes were examined for growth inhibition, and the minimum inhibitory concentration of each plant extract (the minimum concentration inhibiting the in vitro growth of *H. capsulatum var. farciminosum*) was determined. Ketoconazole, at the recommended standard concentration of 0.8 µg/ml, was employed as the positive control, while antifungal-free SDA inoculated with mycelia colony and non-inoculated (sterile) SDA were incubated as negative controls [3, 8].

### Data analysis

Obtained data was entered, checked, and coded into a Microsoft excel spread sheet and analyzed using SPSS version 26 software. Descriptive statistics like percentages were used to express the prevalence of disease and success value of fungal growth inhibition of plant extracts. In all the analysis, a p-value of less than 0.05 was considered statistically significant.

## Data Availability

The original contributions presented in the study are included in the article, and when inquiries are made the raw data supporting the conclusion of this article will be made available by the corresponding author without undue reservation.
